# Interplay between structure and signaling

**DOI:** 10.7554/eLife.99053

**Published:** 2024-06-04

**Authors:** David Biermann, Sebastian Wolf

**Affiliations:** 1 Center for Plant Molecular Biology, University of Tübingen Tübingen Germany

**Keywords:** plant cell wall, pectin, extracellular matrix, rapid alkalinization factors, pectin methylesterases, cell signaling

## Abstract

Modification of pectin, a component of the plant cell wall, is required to facilitate signaling by a RALF peptide, which is essential for many physiological and developmental processes.

**Related research article** Rößling AK, Dünser K, Liu C, Lauw S, Rodriguez-Franco M, Kalmbach L, Barbez E, Kleine-Vehn J. 2024. Pectin methylesterase activity is required for RALF1 peptide signalling output. *eLife* **13**:RP96943. doi: 10.7554/eLife.96943.

The wall surrounding plant cells consists of an elaborate network of proteins and polysaccharides, also known as the extracellular matrix. It provides structural support and defence against pathogens, but it also needs to remain flexible enough so that it can be remodelled in response to environmental cues or during growth. To execute such tasks, a wide range of receptors, pores and channels in the cell wall regulate the movement of and responses to molecules, including hormones, proteins, sugars and RNAs, locally and across cells.

Polysaccharides, such as pectins, are a main component of the cell wall, and they contribute to the wall’s integrity and help to control growth. Pectins are synthesized inside the cell and then transported to the extracellular space in a highly methylesterified form. Enzymes called pectin methylesterases, or PMEs for short, then remove the methyl groups to give rise to negatively charged pectin molecules that can alter the properties of the cell wall (Figure 1A). Researchers have proposed that the methylation status of pectin can be sensed to control growth (Anderson and Kieber, 2020). However, how external signals interact with demethylated pectin molecules in the extracellular matrix is still poorly understood.

**Figure 1.**
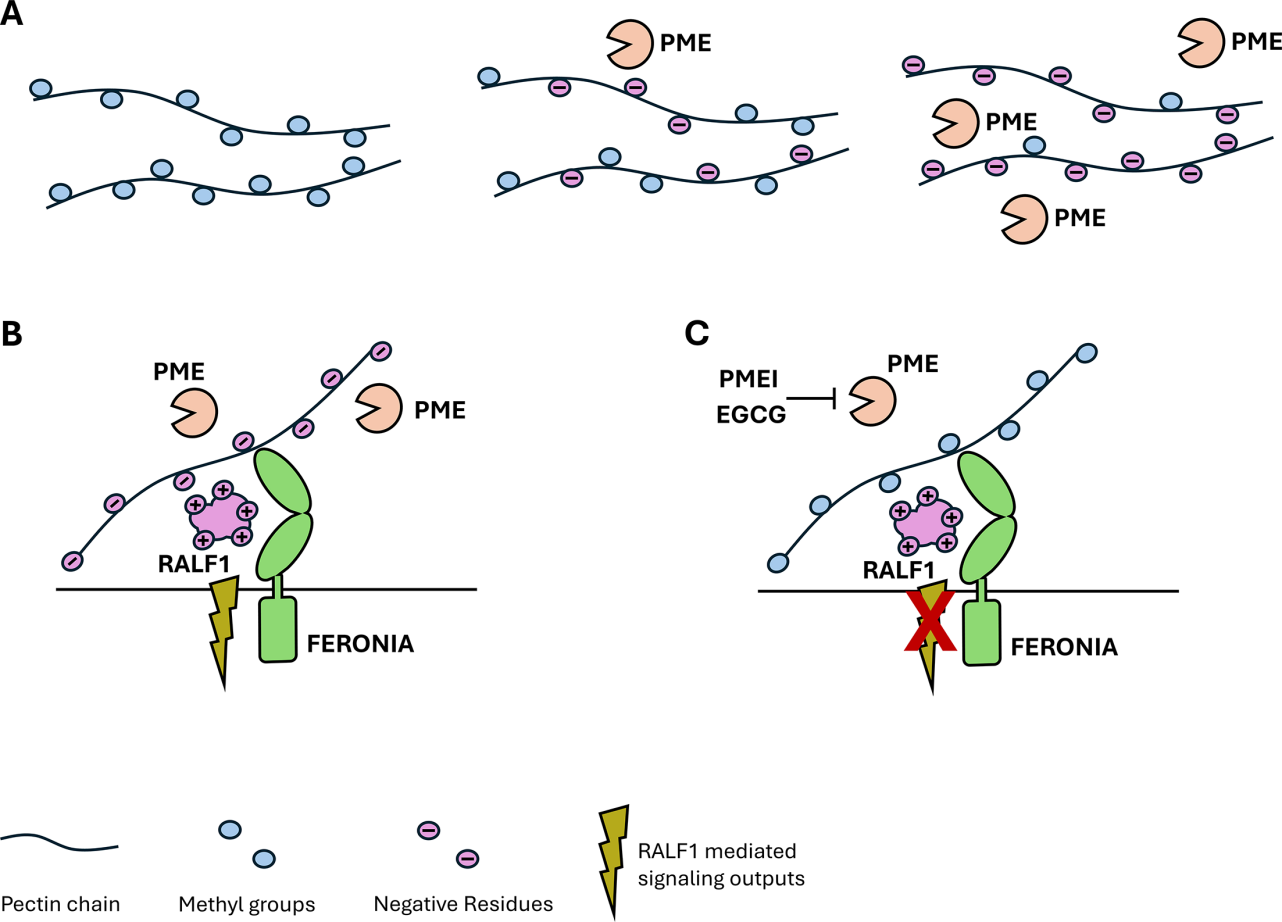
The relationship between polysaccharides, peptides and receptors in the plant cell wall. (**A**) The polysaccharide pectin is a major component of the plant cell wall. Pectins (black strings) are synthesized inside the plant cell and have various methyl groups (blue circles) attached to them before they are transported to the cell wall. Once there, specific enzymes, called pectin methylesterases (PMEs, orange ‘Pac-Man’), remove the methyl groups, which causes pectin to become negatively charged (purple circles). The more PMEs that are present, the more negatively charged the pectins become. (**B**) When PMEs are active, pectin is present in a demethylated, negatively charged form. The positively charged plant peptide RALF1 (large purple structure) can be recognized by its receptor FERONIA (green structure) and trigger specific signaling pathways (green flash). (**C**) Blocking the activity of PMEs by PME inhibitors (PMEI) or pharmacological treatments (epigallocatechin gallate; EGCG), reduces the amount of demethylated pectin and thus inhibits RALF signaling output (crossed out flash).

Now, in eLife, Jürgen Kleine-Vehn, Elke Barbez and colleagues at the University of Freiburg – including Ann-Kathrin Rößling as first author – report new insights into the relationship between pectin and a group of signaling molecules known as rapid alkalinization factors (RALFs; Rößling et al., 2024).

RALF peptides are involved in many physiological and developmental processes, ranging from immune responses to organ growth in plants. One of the most studied RALF receptors is FERONIA, which, upon perceiving RALF, initiates signaling that helps to integrate cues from the environment and contributes to developmental processes in plants (Blackburn et al., 2020). To find out whether pectin modifications affect a specific member of the RALF family, called RALF1, Rößling et al. used genetic and pharmacological approaches to inhibit PMEs in the model plant *Arabidopsis thaliana*. This included overexpressing inhibitors of PMEs and applying a chemical plant compound, called epigallocatechin gallate (EGCG; Jolie et al., 2010; Lewis et al., 2008). They found that both approaches blocked processes controlled by RALF1 signaling, such as inhibition of root growth.

Further experiments showed that the presence of RALF1 led to morphological changes at the cell wall, such as swelling and invaginations of the plasma membrane. These changes were absent when PMEs were blocked. Together, these results hint at a strong link between RALF1 signaling and pectin modification through PMEs (Figure 1B and C).

Next, Rößling et al. set out to confirm that the effects they had observed were due to PMEs directly interfering with RALF1 signaling rather than PME inhibitors or EGCG affecting other signaling pathways. To do this, they tested for consequences that occur immediately after FERONIA perceives and binds to the RALF1 molecule, such as a lower amount of extracellular acidification (also known as alkalinization; Abarca et al., 2021). Indeed, inhibiting PMEs also abolished alkalinization, which suggests that the pectin modification likely affects RALF1 signaling directly.

Another imminent consequence of RALF1 binding to FERONIA is the uptake of the peptide-receptor complex into the cell through a process known as endocytosis (Yu et al., 2020). Indeed, Rößling et al. showed that blocking PMEs, and therefore inhibiting pectin demethylation, also mitigates the endocytosis of FERONIA. Taken together, these results suggest that RALF1 depends on pectin being demethylated in order to be perceived and bind to its receptor FERONIA. Moreover, the researchers showed that RALF1’s positive surface charge enables it to bind to the negatively charged, demethylated pectin, consistent with findings in other studies (Moussu et al., 2023). A mutated version of RALF1 lacking most positive residues was unable to bind to pectin.

The work of Rößling et al. deepens our understanding of peptide signaling at the surface of plant cells. In summary, these findings demonstrate that RALF1 signaling depends on PMEs to demethylate pectin, which is likely because RALF1 and pectin require opposing charges to work together. One interesting possibility raised here is that pectin might act as a reservoir of signaling molecules that are released under certain conditions, reminiscent of the role of the extracellular matrix in some signaling pathways in animals.

More research is needed to fully understand how the interactions between RALF1 and pectin affect the ability of FERONIA to detect RALF1, and how RALF1 signaling itself affects the configuration of pectin. Based on the findings by Rößling et al., RALF1 may only be detected when it is bound to pectin. Several other RALF receptors are also yet to be studied in this context, and it would be interesting to see if their ability to detect members of the RALF family is also linked to the methylation status of pectin.

Structural and biochemical experiments would help to further disentangle the links between cell wall components and peptide signaling. It will also be interesting to see how this process is affected by the large clusters of RALF molecules that have recently been discovered in the extracellular matrix (Liu et al., 2024). Rößling et al. further propose a feedback loop in which PMEs acidify the extracellular space and thereby inhibit their own activity. However, the PMEs also lead to a rise in demethylated pectin, which enables RALF signaling, resulting in cell wall alkalinization. This, in turn, reactivates PMEs. Elucidating the role of pectin modification in these contexts might contribute to a solid model that can explain fascinating signaling processes at the cell surface of plants.
